# Outbreak of Cryptosporidiosis Among Responders to a Rollover of a Truck Carrying Calves — Kansas, April 2013

**Published:** 2014-12-19

**Authors:** Lindsey Martin Webb, Sheri A. Tubach, D. Charles Hunt

**Affiliations:** 1 Kansas Department of Health and Environment

In April 2013, the Thomas County Health Department notified the Kansas Department of Health and Environment’s Infectious Disease Epidemiology and Response section (KDHE) of two cases of cryptosporidiosis among emergency responders to a tractor-trailer rollover. The truck was carrying approximately 350 preweaned Holstein calves. An outbreak investigation was led by KDHE with assistance from the county health department; six cases of cryptosporidiosis were identified among the 15 emergency responders. No additional primary cases with this exposure or secondary cases were identified. Disease was associated with carrying calves (relative risk [RR] = 3.0) and contact with fecal matter (RR = 4.5). The calves were aged <10 days and reportedly suffered from scours (diarrheal disease), which is often caused by *Cryptosporidium spp.* (1), a chlorine-tolerant protozoan parasite. Because of the age of the calves and the conditions at the rollover scene, a high potential existed for fecal contamination and subsequent transmission of *Cryptosporidium*. This outbreak is the first report of both law enforcement and volunteer emergency responders contracting cryptosporidiosis, with transmission of *Cryptosporidium* attributed solely to direct contact with animals and their feces. Human illness resulting from contact with animals during an emergency response might be minimized if 1) all responders are aware of the potential for zoonotic transmission, 2) education is provided on proper animal handling including the use of appropriate personal protective equipment, and 3) responders practice thorough hand hygiene and decontaminate clothing and equipment following contact with feces.

In the early morning of March 10, 2013, a truck carrying approximately 350 Holstein steer calves overturned in a snowstorm near Colby, Kansas. Many of the calves died as a result; many others were scattered outside of the truck. City police officers and county sheriff’s deputies responded to the incident, controlled traffic, and secured the scene. The officers then contacted a towing company and community volunteers with horses and cattle trailers to assist with righting the truck and securing the calves. Because of the very young age of the calves and the injuries and stress resulting from the rollover, most calves that survived the initial impact were unable to walk and had to be carried by responders onto cattle trailers. Responders noted that most of the calves had scours. Deceased calves were loaded into the wrecked truck and towed to the local sale barn. The next day, towing company employees returned to the sale barn and loaded the carcasses onto another truck for shipment to a rendering plant.

Following the report of two cases of cryptosporidiosis in persons who responded to a tractor-trailer rollover involving calves, investigators from KDHE hypothesized that illness might be associated with exposure to calves, fecal contamination at the scene, and returning to a location without electrical power and therefore no hot water to thoroughly wash hands or decontaminate equipment and clothing. A retrospective cohort study was conducted among emergency responders to identify additional ill persons and determine risk factors associated with illness. For this investigation, a probable case was defined as diarrhea (three or more loose or watery stools in 24 hours) and either abdominal cramping, vomiting, or anorexia in an emergency responder within 10 days after the response to the rollover. A confirmed case was defined as an illness that met the definition for a probable case with laboratory evidence of *Cryptosporidium* infection.

KDHE interviewed responders by telephone using an outbreak-specific questionnaire. Fifteen persons participated in the response to this emergency; all were interviewed. Six (40%) respondents were ill and of those, two (33%) had confirmed cases and four (67%) had probable cases of cryptosporidiosis. Fourteen (93%) of the responders were male; all ill persons were male and ranged in age from 17 to 34 years (median = 29 years). Five (33%) responders were law enforcement officers; one became ill. Ten (67%) responders included towing truck employees, the driver of the wrecked truck, and other persons from the community; five were ill. The most common symptoms besides diarrhea were abdominal cramps, anorexia, and weight loss (five [83%] reports each). Five (83%) persons sought medical care.

Although positive rapid antigen test results from stool specimens from two responders prompted this investigation, no additional persons submitted stool specimens. The incubation period ranged from 6 to 8 days (median = 7 days). Among four persons whose illness had resolved by the time of interview, duration ranged from 7 to 13 days (median = 9 days). No deaths or hospitalizations were reported. At the time of the outbreak investigation, no calves were available to be tested for *Cryptosporidium*.

In bivariate analysis, ill responders were statistically more likely than responders who were not ill to have carried calves during the response (RR = 3.0) and to have reported coming into contact with fecal matter (RR = 4.5) (Table). Responders who returned to a location without electrical power following the response were more likely to later become ill than those who returned to a location with power (RR = 4.5); however, this association did not reach statistical significance. No one reported eating any foods during the response; all beverages consumed were contained in sealable plastic bottles and consuming a beverage during the response was not significantly associated with illness (RR = 2.5) (Table).

## Discussion

*Cryptosporidium* transmission is fecal-oral and can occur through ingestion of contaminated recreational water, untreated drinking water, or food, or by contact with infected persons or animals, most notably preweaned calves. Outbreaks caused by *Cryptosporidium* are commonly associated with recreational water, including waterparks and swimming pools, whereas outbreaks associated with zoonotic transmission outside of farm settings are less frequently reported (2). The cryptosporidiosis outbreak described in this report was associated with handling preweaned Holstein calves and coming into contact with calf feces while responding to a tractor-trailer rollover. Six (40%) of the 15 responders became ill with cryptosporidiosis following this response. Occupational outbreaks have been reported in agricultural settings and veterinary schools (3–5). At least one outbreak has been reported among emergency responders following a firefighting response at a location where *Cryptosporidium* was detected in calf fecal specimens as well as in environmental water samples (6). This outbreak is the first report of both law enforcement and volunteer emergency responders becoming infected with *Cryptosporidium* for which only direct contact with animals and their feces was identified as the source of transmission.

Holstein cows are commonly used for milk production; Holstein steers born on dairy farms are sometimes transported to another location to be raised for beef. Very young calves being moved from dairy facilities might be deprived of colostrum and transported with calves from many different farms, which can increase stress and pathogen transmission among calves (7). Scours is common among young calves, and preweaned calves are most likely to be infected with *Cryptosporidium parvum*, a zoonotic species of *Cryptosporidium* that can be transmitted to humans (8). Calves in stressful situations usually experience more severe symptoms of scours associated with an increased shedding of enteric pathogens (7). Before the truck rollover, the calves were transported in crowded conditions over long distances during severe winter weather. Additionally, the calves were reportedly aged <10 days; transporting calves at such a young age might provide more opportunity for pathogen transmission, which can be exacerbated by severe stress. The conditions at the scene of the rollover and the conditions during transport might have led to an increased probability of pathogen transmission. Neither the driver nor the trucking company was cited for any legal violations.

What is already known on this topic?

Cryptosporidiosis is a diarrheal illness caused by the chlorine-tolerant protozoan *Cryptosporidium*. Transmission is fecal-oral and can occur via ingestion of contaminated recreational water, untreated drinking water, or food, or by contact with infected persons or animals, most notably young calves.

What is added by this report?

Two cases of cryptosporidiosis were laboratory diagnosed among 15 persons responding to the rollover of a tractor-trailer carrying approximately 350 calves. An investigation found four additional responders with symptoms meeting a probable case definition. Diarrhea following the exposure was associated with carrying calves and contact with fecal matter. This is the first report of both law enforcement and volunteer emergency responders contracting *Cryptosporidium* for which the mode of transmission was confirmed to be solely zoonotic.

What are the implications for public health practice?

Public health professionals and emergency responders should be aware of the potential for occupational zoonotic transmission during responses to incidents involving animals. Awareness, education, proper hygiene, and personal protective equipment use can prevent transmission of zoonoses during an emergency response.

Contact with livestock, particularly young calves, is a risk factor for zoonotic transmission recognized by health professionals and animal industry workers; however, professional and volunteer emergency responders might be less aware of the potential risk (9). Prior to this rollover response, volunteer responders reportedly were not provided with illness prevention education. Responders did not wear personal protective equipment, but all wore work gloves and heavy outerwear because of the cold weather. Although community members were contacted to provide assistance, no veterinarian was consulted regarding the appropriate care or handling of the calves. A veterinarian could have provided guidance on minimizing transmission of disease while also overseeing humane handling of the animals. The rollover occurred during a snowstorm, and some locations in town did not have electrical power at the time which could have contributed to some persons being unable to appropriately clean or sanitize their clothing and equipment and could have made handwashing less effective or less likely following the response, thus increasing the risk for infection.

This outbreak highlights the need for awareness of zoonotic transmission among those handling calves, including emergency responders. Education of responders is important to prevent future outbreaks of zoonoses that might result from agricultural emergencies (9). Cryptosporidiosis prevention messaging should include instruction on the potential for fecal-oral zoonotic transmission. Education also should be provided on the use of appropriate personal protective equipment (e.g., disposable outer wear, rubber gloves, and rubber boots) during the response and postresponse clean-up. Responders should ensure that all protective clothing is promptly removed and disinfected after handling calves or coming into contact with their feces, followed by thoroughly washing hands with soap and water to prevent infection or recontamination (7). These practices are likely to help reduce fecal-oral exposures during emergency responses involving animals where the potential exists for zoonotic transmission of *Cryptosporidium spp.* and other pathogens.

## Figures and Tables

**TABLE:** Exposures possibly associated with acquiring cryptosporidiosis among responders to the rollover of a truck carrying calves — Kansas, April 2013

Exposure	No. of persons exposed	No. of ill persons exposed	Relative risk	(95% confidence interval)
Carried calves	9	6	3.0	(1.2–7.6)
Contact with fecal matter	8	6	4.5	(1.3–15.3)
Location without power	4	3	4.5	(0.6–33.7)
Beverage during response	8	5	2.5	(0.9–6.7)
